# Short-Term Responses to Fatty Acids on Lipid Metabolism and Adipogenesis in Rainbow Trout (*Oncorhynchus mykiss*)

**DOI:** 10.3390/ijms21051623

**Published:** 2020-02-27

**Authors:** Natàlia Riera-Heredia, Esmail Lutfi, Albert Sánchez-Moya, Joaquim Gutiérrez, Encarnación Capilla, Isabel Navarro

**Affiliations:** Department of Cell Biology, Physiology and Immunology, Faculty of Biology, University of Barcelona, 08028 Barcelona, Spain; natalia.riera@gmail.com (N.R.-H.); esmailroyo@gmail.com (E.L.); alsamo89@gmail.com (A.S.-M.); jgutierrez@ub.edu (J.G.); ecapilla@ub.edu (E.C.)

**Keywords:** eicosapentaenoic acid, linoleic acid, adipocyte, adipose tissue, liver

## Abstract

Fish are rich in n-3 long-chain polyunsaturated fatty acids (LC-PUFA) such as eicosapentaenoic (EPA) and docosahexaenoic (DHA) acids. Due to the increasing use of vegetable oils (VO), their proportion in diets has lowered, affecting lipid metabolism and fillet composition. Rainbow trout cultured preadipocytes were treated with representative FA found in fish oils (EPA and DHA) or VO (linoleic, LA and alpha-linolenic, ALA acids), while EPA and LA were also orally administered, to evaluate their effects on adipogenesis and lipid metabolism. In vitro, all FA increased lipid internalization, with ALA producing the highest effect, together with upregulating the FA transporter *fatp1*. In vivo, EPA or LA increased peroxisome proliferator-activated receptors *ppara* and *pparb* transcripts abundance in adipose tissue, suggesting elevated β-oxidation, contrary to the results obtained in liver. Furthermore, the increased expression of FA synthase (*fas*) and the FA translocase/cluster of differentiation (*cd36)* in adipose tissue indicated an enhanced uptake of lipids and lipogenesis de novo, whereas stable or low hepatic expression of genes involved in lipid transport and turnover was found. Thus, fish showed a similar tissue metabolic response to the short-term availability of EPA or LA in vivo, while in vitro VO-derived FA demonstrated greater potential inducing fat accumulation.

## 1. Introduction

The world population together with fish and seafood consumption have increased over recent years and will continue to rise in the future. Fish products are distinguished for being a rich source of long-chain highly polyunsaturated fatty acids (LC-PUFA) such as eicosapentaenoic (EPA, 20:5n-3) and docosahexaenoic (DHA, 22:6n-3) acids [1,2]. In aquaculture, the maintenance of these levels of n-3 fatty acids (FA) in the flesh is crucial for human health. This can be achieved by providing quality diets assuring the nutritional requirements for each species and an adequate FA profile of the final product [3,4,5]. These diets have been generally based on fishmeal and fish oil (FO) obtained from small pelagic species [6], nevertheless, this could be leading to an unsustainable cycle. Faced with this handicap, the aquaculture industry has invested substantial resources to find alternatives to accomplish these dietary goals. The most economic and sustainable replacements are oils and meals from plant origin. Nonetheless, the use of vegetable oils (VO), presents some disadvantages such as their FA profile, which differs from that of FO, or the presence of anti-nutritional factors. In this sense, the use of highly substituted vegetable diets has shown to increase the occurrence of metabolic alterations [7] and changes in fillet composition [8,9,10,11].

Rainbow trout (*Oncorhynchus mykiss*) is a medium-fat fish nutritionally relevant for the human diet due to its input of high biological value protein, n-3 LC-PUFA as well as vitamins and minerals. In aquaculture production of rainbow trout, the feed was based mostly in fishmeal and FO, but the proportion of the former has been reduced in recent years, with diets nowadays containing around 25% [12] with alternatives as soybean meal [13] and more recently bioprocessed soybean meal [14]. Regarding the FO replacement, soya oil has been used as a substitute of almost 50% of the dietary lipid portion; and mixtures of palm, rapeseed, or linseed oils are currently used in commercial diets [15]. These VO are rich in PUFA n-6 and n-9, principally linoleic (LA; 18:2 n-6) and oleic (OA; 18:1 n-9) acids, except for linseed oil, which contains high amounts of α-linolenic acid (ALA; 18:3 n-3) or palm oil, rich in saturated FA like palmitic (PA; 16:0); but otherwise, they do not contain n-3 LC-PUFA such as EPA and DHA [2,15,16]. This is inconvenient for marine species since there is no transformation of typical FA from VO sources to LC-PUFA as arachidonic acid (AA; 20:4 n-6), EPA or DHA (n-3) due to the low functionality of the responsible enzymes of this pathway [16]. Freshwater species and salmonids can perform these transformations to a certain degree, but in any case, not to ensure a sufficient amount of such FA to maintain the requirements of the species [2].

Furthermore, high dietary VO content can cause metabolic alterations in fish [17,18]. Adipose tissue, as a lipid storage and metabolic organ, has an important role in the regulation of the organism’s energy homeostasis [19]. Growth of this tissue is reached by either hypertrophy or hyperplasia; through increasing cell volume by lipid accumulation, or by formation of new adipocytes from precursor cells, respectively [20]. During the first phase of adipogenesis, transcription factors such as CCAAT/enhancer binding protein alpha (C/EBPα) and beta (C/EBPβ) and peroxisome proliferator-activated receptor gamma (PPARγ) and alpha (PPARα) are activated [21]. Some of these factors stimulate, in turn, the transcription of specific genes related with lipid metabolism (e.g., FA synthase, *fas* and hormone sensitive lipase, *hsl*) and transport, including FA binding proteins, transporters, or translocases (e.g., *fabp11*, *fatp1*, or *cd36*), concluding the adipocyte maturation phase [20]. This differentiation process can be affected by the presence of FA through modifying the expression of the key transcription factors involved, as it has been observed in rodents, with EPA and DHA, natural ligands of PPARγ [22]. Differentiation of fish adipocytes in culture can be also induced by the incubation of precursor cells with specific FA [23,24,25] and lipid storage during the process of adipogenesis can be modified. In this sense, in cultured Atlantic salmon (*Salmo salar*) preadipocytes, and in gilthead sea bream (*Sparus aurata*) bone-derived mesenchymal stem cells, a higher increase in the content of lipid droplets (LD) caused by OA, LA, and ALA, characteristic FA of VO was observed, in comparison with the FA typical of FO, EPA, and DHA [24,25]. Furthermore, FO replacement by VO in gilthead sea bream and Atlantic salmon in vivo, also alters adipose tissue growth enhancing hypertrophy instead of hyperplasia with the consequent dysregulation of lipid metabolism, causing an excess of fat deposition, thus, lowering the quality of the product in terms of fillet yield [18,26].

These altered traits in adiposity are often accompanied by the presence of a fatty liver as seen in gilthead sea bream fed with a diet highly substituted by VO [18], since in fish this tissue is known to also be an important organ of fat accumulation depending on the species. At the same time, the liver is responsible for the distribution of lipids to peripheral tissues having a key role in FA metabolism, therefore being an important organ for the evaluation of the metabolism effects of diet substitution. For all this, expression of genes and proteins involved in adipogenesis, fat metabolism, and lipid transport and accumulation from both adipose and liver tissues have been demonstrated to be good indicators of fish metabolic status [27,28].

In order to study the potential consequences of FO dietary substitution by VO on adipogenesis and lipid homeostasis in rainbow trout, the first most produced freshwater species in Spain, the present study aims first to evaluate EPA, DHA, LA, and ALA treatment in preadipocyte cells in terms of lipid accumulation and gene expression to elucidate the obesogenic potential of FA, especially those from VO. Second, to analyze the effects of a short-term oral administration of EPA and LA on plasma metabolites, and lipid metabolism-related gene and protein expression in adipose tissue and liver to know whether a specific metabolic response in the two main organs of lipid accumulation exists after an increased accessibility of these FA most commonly found in FO or VO, respectively.

## 2. Results

### 2.1. FA Effects in Rainbow Trout Preadipocytes In Vitro

#### 2.1.1. FA Effects on Cell Lipid Content

**Figure 1:**
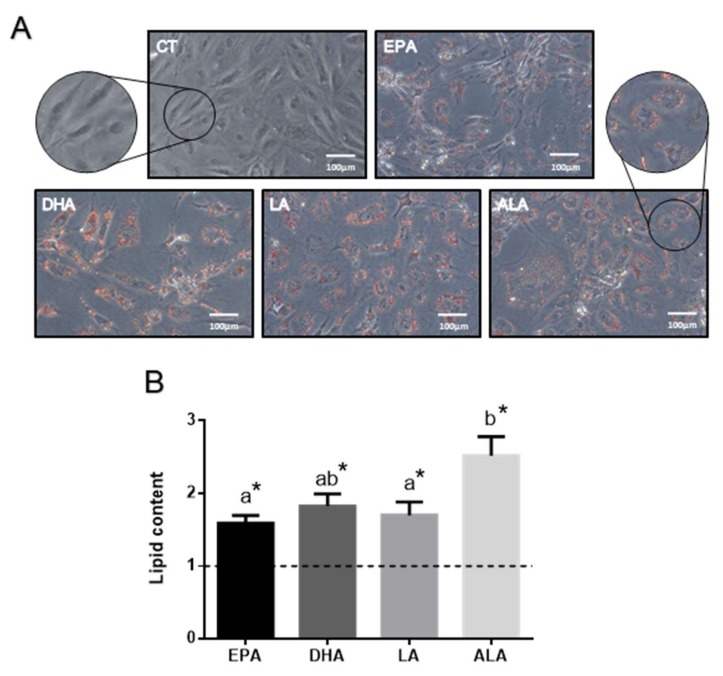
(**A**) Representative phase-contrast images (and 2× enlargements) of rainbow trout preadipocyte cells after staining with Oil red O and (**B**) quantification of lipid content. Cells were treated at day 7 with individual fatty acids (100 µM) or were left untreated as control (dashed line in (B) for 48 h. Data are shown as mean + SEM (*n* = 4). Significant differences (*p *< 0.05) with the control are indicated by asterisks (*) and among treatments by different letters (a,b). CT: control; EPA: eicosapentaenoic acid; DHA: docosahexaenoic acid; LA: linoleic acid; ALA: α-linolenic acid.

The images obtained after (Oil red O) ORO staining of the preadipocytes upon all treatments (EPA, DHA, LA, or ALA 100 µM) showed a higher cell lipid accumulation than those in control (CT) cells (Figure 1A). In addition, changes on cellular morphology in response to the treatments were observed, the cells becoming more rounded with an enlarged cytoplasm while losing the fibroblastic shape, indicating the initiation of the differentiation process.

Lipid content analyses confirmed image observation, showing that cells treated with either one of the FA contained significantly higher intracellular lipids compared to the control cells without FA (*p* < 0.01) (Figure 1B). Among the treatments, ALA induced the highest accumulation of neutral lipids in the preadipocytes, showing significant differences with EPA and LA (*p *< 0.05), but not DHA.

#### 2.1.2. FA Effects on Adipogenesis and Lipid Metabolism-Related Preadipocyte mRNA Levels

To further determine the effects of the individual FA on rainbow trout preadipocytes, gene expression of the main genes involved in the adipogenic process and lipid metabolism were analyzed (Table 1A). The mRNA levels of *pparg* and *cebpa*, two key transcription factors of adipogenesis, did not change with the treatments except after EPA incubation, which induced a significant downregulation compared to the control condition (*p* < 0.05). Regarding other transcription factors and lipid metabolism-related genes, significant differences were not observed neither with respect to the control or among the different FA-treated cells, except for the FA transporter protein *fatp1* that showed a significant increase in mRNA levels after incubation with the two FA typical of VO (LA and ALA) compared to the control condition (*p* < 0.001) and DHA (*p* < 0.03).

Moreover, the transcript levels of those genes were also studied in response to the presence of different FA combined in pairs (Table 1B). As in individual treatments, significant differences were not found for most genes, except for *pparg*, liver X receptor (*lxr*), and *fatp1*. Specifically, *pparg* was significantly downregulated by all treatments compared to the control condition (*p* < 0.000), as also the combination of EPA and DHA caused a reduction in *lxr* mRNA levels (*p* < 0.001). Finally, the combinations of LA with either EPA or ALA showed a significant increase on *fatp1* mRNA levels when compared to the control cells (*p* < 0.01) and those incubated with the combination of EPA plus DHA (*p* < 0.005).

### 2.2. FA Effects in Rainbow Trout In Vivo

#### 2.2.1. FA Effects on Plasma Metabolites

**Figure 2:**
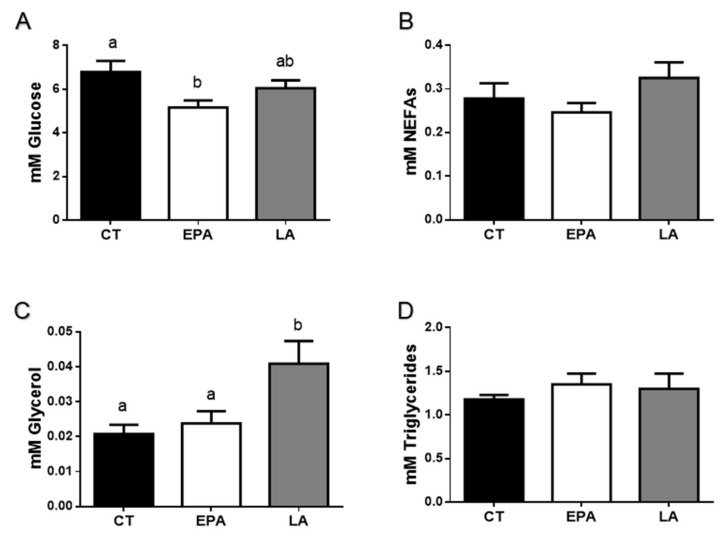
Rainbow trout (**A**) glucose, (**B**) NEFAs, (**C**) glycerol, and (**D**) triglyceride plasma levels in rainbow trout at 6 h after being force-fed with EPA, LA, or vehicle without fatty acid as control. Data are shown as mean + SEM (*n* = 10). Significant differences (*p* < 0.05) among treatments are indicated by different letters (a,b). CT: control; EPA: eicosapentaenoic acid; LA: linoleic acid; NEFAs: non-esterified fatty acids.

Representative FA of the two oil sources, fish or vegetables, EPA and LA, respectively, were selected to study the effects in vivo in rainbow trout after a gavage procedure. Six hours after FA administration, plasma analyses showed significant differences in glucose (Figure 2A) and glycerol (Figure 2C), but not in NEFA (Figure 2B) or triglyceride (Figure 2D) levels. EPA treatment caused a diminution in glucose levels compared to the control fish (*p* < 0.05), while LA increased the concentration of glycerol when comparing to control and EPA-treated animals (*p* < 0.05).

#### 2.2.2. FA Effects in Adipogenesis and Lipid Metabolism-Related Genes and Protein Levels in Adipose Tissue

**Figure 3:**
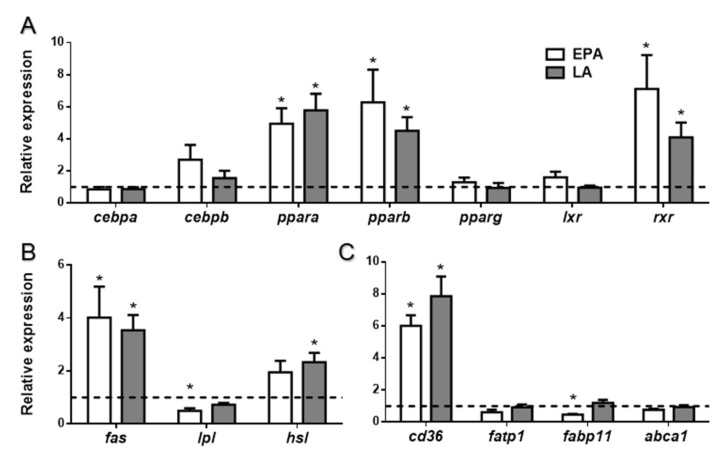
Relative gene expression of (**A**) transcription factors, (**B**) lipid metabolism enzymes, and (**C**) lipid transporters in adipose tissue of rainbow trout at 6 h after being force-fed with EPA, LA, or vehicle without fatty acid as control (dashed line). Data are shown as mean + SEM (*n* = 7–9). Significant differences (*p* < 0.05) with the control are indicated by asterisks (*) and among treatments by different letters. EPA: eicosapentaenoic acid; LA: linoleic acid.

The transcription factors *ppara*, *pparb*, and retinoid X receptor (*rxr*) were significantly upregulated by EPA and LA when compared to the control fish (*p* < 0.05), but differences between the two FA treatments were not found (Figure 3A). Contrarily, expression of *cebpa*, *cebpb*, *pparg*, and *lxr* remained similar to the control fish. Regarding the lipid metabolism-related enzymes analyzed, *fas* expression was increased in rainbow trout in response to the two FA (*p* < 0.05), *hsl* was upregulated only by LA treatment (*p* < 0.01), while lipoprotein lipase (*lpl*) was contrarily downregulated but in this case after EPA treatment (*p* < 0.05) (Figure 3B). Concerning FA transporters, the FA translocase/cluster of differentiation *cd36* was significantly upregulated after the administration of both FA compared to the control condition (*p* < 0.001) (Figure 3C). Moreover, the FA binding protein *fabp11* showed a significant decrease with respect to the control, in the fish treated with EPA (*p* < 0.01).

**Figure 4:**
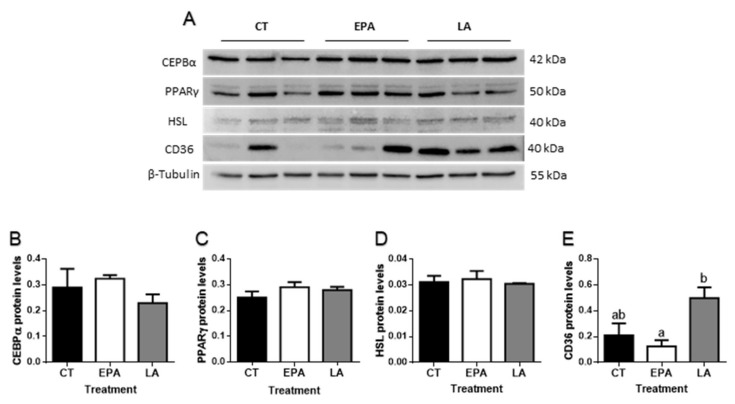
(**A**) Representative Western blots of the transcription factors CEBPα and PPARγ, the enzyme HSL, the fatty acid translocase CD36, and β-tubulin in adipose tissue of rainbow trout at 6 h after being force-fed with EPA, LA, or vehicle without fatty acid as control. Quantification of the protein levels of (**B**) CEBPα, (**C**) PPARγ, (**D**) HSL, and (**E**) CD36 normalized to β-tubulin. Data are shown as mean + SEM (*n* = 6). Significant differences (*p* < 0.05) among treatments are indicated by different letters (a,b). CT: control; EPA: eicosapentaenoic acid; LA: linoleic acid.

Effects on the levels of representative protein members of each cluster of genes studied were also analyzed (Figure 4A). Differences were not found for the two transcription factors CEBPα (Figure 4B) and PPARγ (Figure 4C), neither for the enzyme HSL (Figure 4D). Concerning the FA transporters, CD36 levels increased in rainbow trout after LA treatment, but only significantly in comparison to the EPA-treated fish (*p* < 0.05) (Figure 4E).

#### 2.2.3. FA Effects in Lipid Metabolism-Related Genes and Protein Levels in Liver

**Figure 5:**
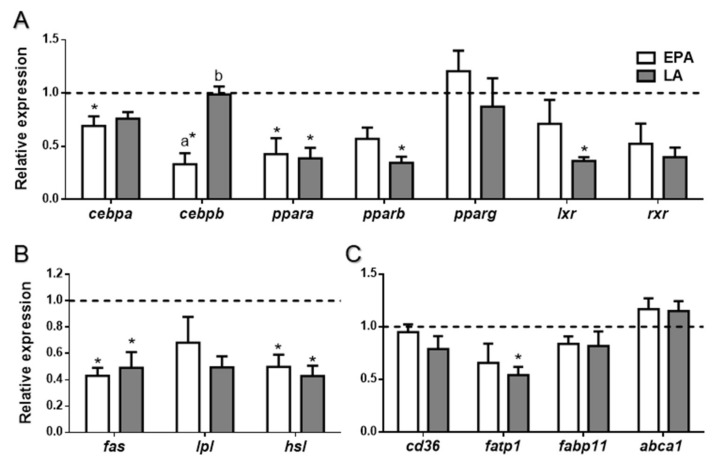
Relative gene expression of (**A**) transcription factors, (**B**) enzymes, and (**C**) lipid transporters in liver of rainbow trout at 6 h after being forced-fed with EPA, LA, or vehicle without fatty acid as control (dashed line). Data are shown as mean + SEM (*n* = 6–8). Significant differences (*p* < 0.05) with the control are indicated by asterisks (*) and among treatments by different letters (a,b). EPA: eicosapentaenoic acid; LA: linoleic acid.

Expression of the same clusters of genes as in adipose tissue was determined in liver. Regarding transcription factors, *ppara* was the only gene whose expression was significantly downregulated by both FA treatments (*p* < 0.02). LA also significantly downregulated *pparb* and *lxr*, with respect to the control animals (*p* < 0.01), whereas EPA significantly diminished the expression of *cebpa* and of *cebpb* (*p* < 0.01), the latter also compared to LA-treated fish (*p* < 0.01) (Figure 5A). Concerning the transcript levels of the enzymes related to lipid metabolism, *fas* and *hsl* were significantly downregulated in rainbow trout after the two FA treatments when compared to the control condition (*p* < 0.05 and *p* < 0.0001, respectively) (Figure 5B). Moreover, among the lipid transporters analyzed, only *fatp1* expression was significantly reduced in fish after LA treatment (*p* < 0.05) while the rest of the genes remained unaltered (Figure 5C).

**Figure 6:**
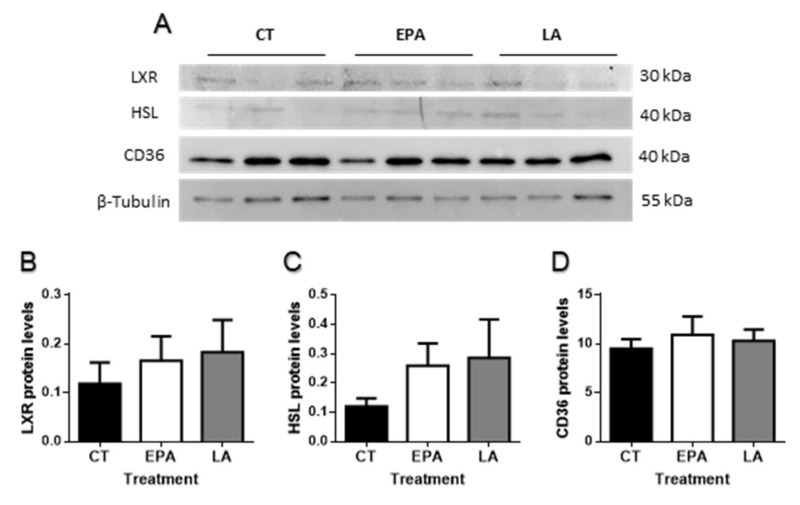
(**A**) Representative Western blots of the transcription factor LXR, the enzyme HSL, the fatty acid translocase CD36, and β-tubulin in the liver of rainbow trout at 6 h after being force-fed with EPA, LA, or vehicle without fatty acid as control. Quantification of protein levels of (**B**) LXR, (**C**) HSL, and (**D**) CD36 normalized to β-tubulin. Data are shown as mean + SEM (*n* = 6). Significant differences (*p* < 0.05) among treatments were not found. CT: control; EPA: eicosapentaenoic acid; LA: linoleic acid.

Finally, differences were not found in the protein levels (Figure 6A) of either LXR (Figure 6B), HSL (Figure 6C), or the FA translocase CD36 (Figure 6D).

## 3. Discussion

Research in aquaculture has been investigating new sustainable strategies to face the lowered pelagic stocks to produce fish feeds, using VO to substitute FO. The different dietary FA profiles in these oils have shown to differentially affect fish lipid metabolism and adiposity [17,25,28]. In this context, the relevance of testing the direct effects of specific FA, in vitro as well as in vivo, must be taken into account to obtain a general view of the importance of FO substitution in fish lipid metabolism. To this end, this study focused on investigating the effects of FA typical from FO and those most commonly found in VO, on rainbow trout adipogenesis and lipid metabolism regulation, using as experimental models an in vitro culture of preadipocytes and an in vivo administration experiment by gavage. The main objective was to evaluate the potential differences caused by these FA in the process of adipogenesis and preadipocyte metabolism, as well as to analyze in vivo the short-term transcriptional response of key tissues involved in lipid homeostasis after an increase in ingested FA.

Cell morphology changes and increased lipid accumulation (both as indicative of adipocyte differentiation) were observed after incubation with the different FA, demonstrating for the first time in rainbow trout preadipocytes that the presence of a single FA, without any other lipid or hormonal component, is able to induce precursor cell differentiation into an adipocyte-like phenotype. Indeed, it has been reported that the presence of lipids in the media is critical to induce adipogenesis in fish cells [29,30,31,32], which has been also observed in avian preadipocytes [33]. Accordingly, in the present study, the cells began to accumulate lipids in response to all the FA treatments after 48 h, although ALA produced the greatest effect; overall, suggesting that this FA may stimulate, more than the others, uptake and fat depot in these cells (i.e., adipocyte differentiation). Similarly, in mature salmon adipocytes it was observed that FA commonly found in VO such as OA, are able to induce more lipid accumulation than FA characteristic of FO [25], as previously reported in mammalian 3T3-L1 adipocytes [34]. In this context, this effect of FA from VO could lead in vivo to dysregulation of lipid metabolism, with the induction of adipose tissue growth by hypertrophy. This was observed in Atlantic salmon and gilthead sea bream fed diets where FO was highly replaced by VO, resulting in an excess or alteration of fat deposition [15,23]. Moreover, the same enhanced lipogenic capacity of FA abundant in plant oils (e.g., LA) compared to those from FO in gilthead sea bream bone-derived mesenchymal stem cells has been recently reported [24], thus, suggesting the potential of such FA to induce both adipogenesis as well as mature adipocyte hypertrophy.

In rainbow trout, after an initial characterization of the adipogenic process [29], a complete transcriptional profile was established demonstrating the coordinated expression of adipogenic genes during the phases of proliferation and differentiation [35]. More recently, a transient upregulation of key genes after 24 h of induction of adipogenesis, followed by a decrease in their expression has been described [36]. Nonetheless, information is limited regarding the activation of preadipocytes in response to specific FA or their combinations after short incubations.

In the present study, the expression of the transcription factors *cebpa* and *pparg* was downregulated by EPA, and *pparg* mRNA levels were also decreased by all the paired FA combinations. These results are in accordance with the anti-adipogenic effect of EPA and DHA observed in several in vitro experiments performed in 3T3-L1 cells [34,37] or fish adipocytes [38]; although other authors have also demonstrated absence of or even a pro-adipogenic effect of these two FA in the same mammalian cell line model [39,40,41]. Nevertheless, these differences could be explained by variable incubation times or cell conditions among experiments [42]. Particularly, the stage of cell differentiation and previous basal levels of expression seem to affect the effects of FA. In this sense, in Atlantic salmon mature adipocytes a significant reduction in the expression of *cebpa* after EPA incubation was also shown [38]. On the other hand, in undifferentiated bone-derived cells from gilthead sea bream, 6 h of EPA treatment increased the expression of *pparg* when compared to LA and ALA [24]. Studies using Atlantic salmon and rainbow trout preadipocytes have demonstrated that possibly these cells are destined early to be adipocytes due to they are already expressing *pparg* before the induction of differentiation [36,43]. This fact could help to explain the decrease of expression of this transcription factor in our cell model, as a readjustment of its regulation during the induction of the adipogenic process. Nevertheless, the effect of FA modulating adipogenesis and lipid accumulation in mammals, as well as in fish, is very complex and not completely understood yet.

Moreover, in our study, the expression of other adipocyte differentiation-related genes, specifically those that code for key enzymes such as *fas*, *hsl*, or *lpl*, remained stable or low, as observed after adipogenesis induction in gilthead sea bream preadipocytes [44]. In fact, FA in the culture media seem to provoke a downregulated expression of *fas*, possibly through a negative feedback mechanism [24,44]. In agreement with our results, *lpl* and *hsl* gene expression levels did not show significant changes during adipocyte differentiation when the whole transcriptional profile of this process was analyzed [35]. The present results could also be suggesting a maintenance of the metabolic turnover through a balance between lipolysis and lipogenesis, a characteristic of the adipocyte to avoid lipotoxicity caused by FA [45].

Concerning the genes involved in the uptake and transport of FA, only the mRNA levels of *fatp1* increased significantly in the cells upon treatment, specifically in response to LA, ALA, or the combinations containing either one of these FA. These data indicated that this transporter is rapidly activated in the presence of extracellular lipids and should have an important role in the uptake of FA from the environment in fish preadipocytes in agreement with previous studies [25,46]. All in all, the FA characteristic of VO (LA and ALA) apparently demonstrated more potential to upregulate this FA transporter. This was accompanied as mentioned above by a higher effectiveness in stimulating lipid uptake and accumulation; as previously reported in Atlantic salmon preadipocytes and in bone-derived cells from gilthead sea bream [24,38].

To further increase knowledge on FA effects in rainbow trout, after the in vitro approach, we used EPA and LA in a short-term in vivo trial. We selected EPA as being one of the main representatives of LC-PUFA found in FO, and LA, since it is commonly found in VO and at the same time is not a precursor of EPA, thus, avoiding potential overlapping effects.

In this context, in rainbow trout it was shown that the oral administration of FO or its main FA (EPA and DHA) by gavage or intraperitoneally, improves the control of glycemia [47,48]. Similarly, in mammals, some studies demonstrated that n-3 LC-PUFA improves insulin resistance and the secretion of this hormone [49,50]. In our study, only EPA administration showed a decrease in glucose plasma levels confirming that this protective role could be characteristic of n-3 LC-PUFA. Furthermore, LA-treated fish presented increased glycerol plasma levels when compared to those animals that were administered vehicle or EPA treatments. In mammals, increased plasma glycerol has been associated with a mild hypoglycemia besides being a possible sign of adipose tissue lipolysis [51]. Nevertheless, no effect was observed in circulating triglycerides after the different FA treatments. Despite being a short-term study, these results are in concordance with a long-term dietary VO substitution trial in gilthead sea bream, where changes in triglycerides and glucose levels were not found [18]. In contrast, triglycerides levels in plasma were lower in mice fed diets supplemented with n-3 PUFA compared to ones fed diets with n-6 PUFA [52]. Thus, the impact of the increase in FA disposal in rainbow trout plasma parameters was relatively small.

These mild changes in circulating metabolites could be reflecting quick adjustments in adipose tissue and liver energy balance. In adipose tissue, lipid metabolism homeostasis and adipogenesis are partially controlled by PPARs, which can act as lipid sensors contributing to homeostasis in circumstantial physiological conditions, when activated by PUFA or eicosanoids [53,54]. In our study, the administration of EPA and LA induced an increase in the expression of the transcription factors *ppara* and *pparb*, suggesting an activation of FA oxidation to provide energy to the adipocyte. On the other hand, differences were not found either in gene or protein expression of PPARγ that besides regulating adipogenesis also controls genes involved in lipogenesis [29,55]. These results were similar to those found in vitro, except for EPA and the FA combinations, which caused a decrease in *pparg* transcript expression, suggesting overall that the cellular system seems to be more sensitive to specific FA availability, in this case probably delaying cell development. Nevertheless, *fas* showed a clear upregulated expression in adipose tissue from both FA force-fed rainbow trout, indicating increased lipogenesis de novo, suggesting a stimulation of lipid anabolism with the FA treatments in vivo. On the other hand, *lpl* was regulated in a different way by the two FA, since its expression was reduced significantly by EPA, but not by LA. The regulation of *lpl* could be thus FA specific as shown in *Pagrus major*, where diets supplemented with OA and EPA produced a diminished expression of adipose tissue *lpl*, but not after supplementation with OA and LA [56]. Therefore, the type of FA present in the feed influences adipose tissue* lpl* expression, as it occurs in mammals [57,58,59]. Moreover, *hsl* mRNA levels were upregulated after LA administration, although this result was not correlated with protein levels, where differences were not found. These data are in agreement with a study in isolated adipocytes from gilthead sea bream fed with vegetable diets, where upregulated expression of *hsl* together with an increase in HSL activity was reported [18]. These results suggested enhanced intracellular lipolysis as demonstrated also in mammals [60,61,62], whereas in our in vitro model, the gene expression of these lipid metabolism-related enzymes remained similar in all conditions when compared to the control. This different response in cultured cells could be because the treatments were performed at an early stage of adipocyte differentiation. Altogether, in vivo, the global response of adipose tissue to the presence of FA seems to be a general activation of lipid turnover, (i.e., lipolysis and lipogenesis), quite independently of the type of FA fed.

On the other hand, the general downregulated response of the liver in terms of gene expression was different from that of adipose tissue, in agreement with the fact that the expression of many of the genes related to lipid metabolism are known to be regulated in a tissue specific manner [63]. For instance, mRNA levels of *cebpa* and *cebpb* were significantly downregulated by EPA treatment in this tissue, and a decreased expression in *ppara* and *pparb* was also observed after administration of both FA. These results could indicate a reduction in FA metabolism in the liver due to treatments, as shown in a study with similar experimental conditions [46,47]. Expression of *lxr*, a transcription factor related to the lipogenic pathway in liver [64], was decreased in our study by LA treatment, together with a significant downregulation of *fas* by the two FA, suggesting a decrease in lipogenesis, contrary to what was found in adipose tissue. In fact, several studies have shown *fas* decreased expression levels in rainbow trout both after FA incubations in hepatic cells [65] and intraperitoneal injection of OA [47], or after administration of diets with increased content of n-3 and n-6 PUFA in mice [52]. In addition, the reduced *hsl* mRNA levels could indicate a decrease in lipolysis, thus overall indicating decreased hepatic activity in response to FA.

Regarding FA transporters and binding proteins in adipose tissue, the upregulation observed in *cd36* gene expression as a result of the two FA treatments (i.e., higher tissue availability), suggests an increased uptake of FA into the adipocyte [66]. This change was not observed in our in vitro model, supporting that other systemic factors might be involved in the regulation of *cd36* mRNA levels, although *fatp1* expression indeed increased after LA and ALA adipocyte incubations, but was not modified after in vivo FA administration. These responses suggest a possible differential role of the two transporters, which have been also described to present different expression patterns among tissues [67,68]. Besides, differences among the effects observed after FA treatments in in vitro or in vivo experiments are commonly found; in fact, a systemic response may involve additional factors that can modulate the effects observed in vitro [69]. In any case, only LA succeeded in increasing CD36 protein levels in adipose tissue when compared to EPA, which could evidence a probably different post-transcriptional regulation of this transporter depending on the FA present and reinforcing the higher potency of FA from vegetal origin to promote lipid uptake.

On the contrary, differences were not found in FA transporters’ expression in the liver, except for the downregulation of *fatp1* by LA treatment. This result is in concordance with a study, where the hepatic FA transporters analyzed remained unchanged after treatment [47]. All this could indicate also a different FA dynamism between the two tissues analyzed and, thus, lipid internalization could have finished in the liver at the selected time of sampling. This would help to explain the different observed patterns in gene expression in both tissues, suggesting a general activation of lipid metabolism in adipose tissue and a depression in liver.

In summary, the FA tested increased lipid accumulation in cultured adipocytes from rainbow trout, especially ALA, accompanied by an increase in the mRNA levels of the FA transporter *fatp1*. Thus, in vitro results support a greater potential capacity of FA characteristic of VO to induce fat accumulation in the adipocyte, confirming that VO included in aquafeeds could potentiate greater adiposity and their consequently negative effects in fish health. The short-term administration of EPA or LA in vivo appeared to activate lipolytic and lipogenic pathways in the adipocyte simultaneously, as a response to a rapid increase in available FA, although without clear differences between the two FA. The capacity of the different FA to be accumulated in the adipose tissue in this type of experiment cannot be distinguished, but it was clear that the FA translocase CD36 seems to be a key FA sensor in a situation of increased FA tissue availability. Changes in the mRNA levels of the genes analyzed in the liver suggested a decrease in fat metabolism, altogether indicating that tissue specific FA dynamics could be responsible for the different responses. Overall, the present results provide valuable information on rainbow trout lipid metabolism that may help the design of FA profile in feed formulations to ensure an optimal degree of fish adiposity.

## 4. Materials and Methods

### 4.1. Animals and Ethics Statement

Adult rainbow trout (*O. mykiss*) of approximately 250 and 124.7 ± 5.3 g in weight for the in vitro and in vivo experiments, respectively, were obtained from the fishery “Troutfactory S.L.” (Lleida, Spain) and were acclimated to the facilities at the Faculty of Biology of the University of Barcelona for two weeks. The choice of fish this size was, in each case, to have enough adipose tissue to perform the in vitro cultures and to appropriately administer the FA by gavage in the in vivo experiment, but also to avoid having gonads developed since we have found this to negatively affect the proper development of the cells in culture (unpublished observations). Fish were kept in 400 L fiberglass tanks under a 12 h light/12 h dark photoperiod at 15 °C and fed ad libitum twice daily with a commercial diet (Optiline-sf, Skretting, Burgos, Spain; 40% protein, 25% lipid and 19 MJ/kg digestible energy) before performing any of the procedures. Then, fish were fasted 24 h before both the in vitro and in vivo experimental manipulations in order to avoid contamination from the gastrointestinal tract during the adipose tissue extraction for cell culture and to have the gut empty during the gavage procedure. Before sacrifice by cranial concussion, fish were anesthetized with ethyl 3-aminobenzoate methanesulfonate (MS222, E10521, Sigma–Aldrich, Tres Cantos, Spain). All animal handling procedures complied with the Guidelines of the European Union Council (86/609/EU) and were approved by Ethics and Animal Care Committee of the University of Barcelona, following the regulations and procedures established by the Spanish and Catalan governments (permit number OB35/17).

### 4.2. Primary Culture of Adipocyte Cells and FA Treatment

All cell culture reagents were purchased from Sigma–Aldrich (Tres Cantos, Spain) and Life Technologies (Alcobendas, Spain). All plastic materials were obtained from Nunc (LabClinics, Barcelona, Spain). Cells were isolated (date of collection: 07/2017) from a pool of white adipose tissue from 6–7 rainbow trout and cultured according to the previously established procedure by [29]. The dissected fat pad was washed with Krebs-HEPES buffer (pH 7.4), and digested for 1 h with type II collagenase 130 UI/mL in Krebs-HEPES buffer containing 1% bovine serum albumin at 18°C. The cell suspension obtained was filtered (100 μm) and centrifuged (700 *g*, 10 min). The cells were counted and seeded in plates with 1% gelatin at a density of 2 × 10^4^ cells/cm^2^ in growth media, containing Leibovitz’s L15 medium supplemented with 10% fetal bovine serum (FBS) and 1% antibiotic/antimycotic solution. To perform the lipid quantification assay, the cells were seeded in 24 well plates, and in six-well plates for the gene expression analyses. The FA were applied in the same growth medium, alone or in combination, once confluence was reached (day 7), and the duration of the treatments was 6 h for gene expression analyses, and 48 h to quantify lipid accumulation. Six independent cultures were performed (*n* = 6), but in all cases, two wells within each culture were used for each experimental condition as internal duplicates. The FA (EPA, DHA, LA, and ALA) were obtained from Cayman Chemical Company (Ann Arbor, MI, USA), first dissolved in ethanol and used at a final concentration of 100 µM both individually and in all tested combinations unless stated otherwise. The doses were selected according to previous studies in adipocytes reviewed in [42], and considering that in in vitro trials, supraphysiological concentrations are usually employed to obtain clearer responses. A control condition without FA but vehicle (ethanol) was also performed. The final concentration of ethanol was below 1% to avoid causing negative effects in cell viability as tested in preliminary assays and in agreement with other studies [24,41].

### 4.3. Oil Red O Staining

To evaluate differentiation of precursor cells into mature adipocytes, intracellular neutral lipid accumulation was analyzed by Oil Red O staining as explained in a previous study in the group [31]. Briefly, cells were fixed with buffered formalin 10% for 1 h, rinsed in PBS, stained in Oil Red O solution, and lipid content was determined by measuring absorbance at 490 nm (Infinite 200, Tecan, Männedorf, Switzerland). Afterwards, Comassie blue staining was performed and the dye extracted to determine protein levels. Thus, quantification of cell lipid content was calculated as the absorbance measured at 490 nm divided by the read at 630 nm corresponding to the protein content [31]. Data are presented as fold change relative to the control (*n* = 3–4). Pictures of the stained cells were obtained with a Zeiss Axiovert 40 C inverted research grade microscope (Carl Zeiss Inc., Oberkochen Germany) equipped with a Canon EOS 1000D digital camera. Magnification 20×.

### 4.4. In Vivo FA Treatment by Gavage

To evaluate the effect of selected FA by gavage, the following treatments were used (date of collection: 27/07/2018): EPA, LA, and vehicle as control, 30 fish (*n* = 10) were administered manually using 2 mL syringes coupled to a cannula large enough to reach the stomach as previously described [47]. The FA were obtained from Cayman Chemical Company (Ann Arbor, MI, USA) and were dissolved in ethanol as described in Section 4.2. The administration dose was 20 mg of FA per kg of animal, force-feeding the fish with 1 mL of solution per 100 g. The dose used was calculated taking into account previous FA administration studies, daily usual dietary FA intake, and postprandial plasma and liver levels of FA in rainbow trout, in order to mimic a post-feeding situation [70,71]. The stock solution was diluted with tank water [46]. Additionally, blue food coloring was added to the solution to confirm correct administration. The solution for the control animals, without FA, contained the same ethanol concentration as the experimental solutions, tank water, and food coloring.

After 6 h of the force-feeding procedure, fish were anesthetized with MS222 and blood was extracted from the caudal vein. Fish were sacrificed by cranial concussion, and samples of adipose tissue and liver were obtained, snap frozen in liquid nitrogen and stored at −80 °C.

### 4.5. Plasma Analyses

Blood was centrifuged 10 min at 5000 rpm to extract plasma. Plasma samples (*n* = 10) were analyzed using commercial enzyme kits: glucose (Monlab, Barcelona, Spain), non-esterified FA (NEFAs, Wako Chemicals GmbH, Neuss, Germany), and triglycerides and glycerol (Sigma-Aldrich, Tres Cantos, Spain).

### 4.6. RNA Extraction and cDNA Synthesis

In adipocyte cultures (*n* = 6) the cells were lysed with a cell scraper and TRI Reagent (Applied Biosystems, Alcobendas, Spain) in a total volume of 1 mL per each two wells [31]. For tissue samples, 100 mg of adipose tissue (*n* = 9) and 50 mg of liver (*n* = 8) were homogenated in 1 mL of TRI Reagent, using Precellys Evolution (Bertin Instruments, Montigny-le-Bretonneux, France) [28]. Total RNA was extracted according to the manufacturer’s recommendations, dissolved in DEPC-treated water (RNase-free), quantified using a NanoDrop 2000 spectrophotometer (Thermo Scientific, Alcobendas, Spain), and stored at −80 °C. To eliminate any residual genomic DNA, total RNA (1 μg) was treated with DNase I (Invitrogen, Alcobendas, Spain) and converted into cDNA using the Transcriptor First Strand cDNA Synthesis Kit (Roche, Sant Cugat del Valles, Spain), following the manufacturer’s instructions.

### 4.7. Quantitative PCR Analyses

Key genes implicated in adipogenesis and energy metabolism regulation were analyzed by real-time quantitative PCR (qPCR) following the procedure previously described in a previous study in the group [72]. The genes evaluated comprise the following (Table 2): the transcription factors or nuclear receptors*: cebpa, cebpb, ppara, pparb, pparg*, *lxr*, and *rxr*; the enzymes: *fas*, *lpl*, and *hsl*; and lipid transporters: *cd36*, *fatp1*, *fabp11*, and ATP-binding cassette transporter *(abca1*). Relative expression levels of the target genes were determined by the Pfaffl method [73] using correction for primer efficiencies and normalizing the quantification cycle (Cq) value of each gene registered during the annealing step to that of the most stable reference genes on each condition and determined using the CFX Manager Software (Bio-Rad, El Prat de Llobregat, Spain). Reference genes for the in vitro experiment were elongation factor 1 alfa (*ef1a*) and ubiquitin (*ub*), for adipose tissue were beta-actin (*b-actin) *and* ef1a*, and for liver were* 18s *and* ef1a.*

### 4.8. Protein Extraction and Western Blot Analysis

To perform extraction and Western blot analyses and subsequent quantification of the proteins of interest, previously published protocols were followed [74,75]. Briefly, to perform protein extraction, 100 and 50 mg of adipose tissue and liver were used, respectively (*n* = 6). To homogenize the tissues 350 µL of RIPA (supplemented with proteases and phosphatases) was added to the samples and the Precellys Evolution coupled to a Cryolys cooling system (Bertin Instruments, Montigny-le-Bretonneux, France) was used. The supernatants were collected after a centrifuge of 30 min and stored at −80 °C. Protein quantification was done by the Bradford method and, 12 µg of protein was subjected to electrophoresis (SDS-PAGE) on 15% polyacrylamide gels (125 V for 1 h 30 min). After overnight transfer to a PVDF-FL membrane, a staining with Revert^TM^ Total Protein Stain (LI-COR Inc. Biotechnology, Lincoln, NE, USA) was performed to confirm that similar amounts of transferred proteins were on each lane. Subsequently, membranes were washed and blocked with Odyssey^®^ Blocking Buffer (LI-COR Inc. Biotechnology, Lincoln, NE, USA) and then incubated with the respective antibody: anti-CEBPα (#sc-61), anti-PPARγ (#sc-7196), and anti-LXR (#sc-1202) from Santa Cruz Biotechnology Inc. (Dallas, TX, USA) and anti-HSL (#4107S), anti-CD36 (#D8L9T), and anti-β-tubulin (#2146S) from Cell Signalling Technology Inc., (Danvers, MA, USA). After washing, the membranes were incubated with an IRDye secondary antibody (LI-COR Inc. Biotechnology, Lincoln, NE, USA). The bands were visualized by infrared fluorescence using the Odyssey Imaging System (LI-COR Inc. Biotechnology, Lincoln, NE, USA) and quantified using the Odyssey Infrared Imaging System software (version 1.2; Application Software). The signal band of each protein of interest was normalized to the signal of β-tubulin.

### 4.9. Statistical Analyses

First, data normality and homoscedasticity were assessed using the Shapiro–Wilk and Levene’s tests, respectively. Comparison between two groups (each experimental treatment versus the control) was assessed by independent samples’ Student’s *t*-test. For multiple mean comparisons (among FA treatments) of normal distributed data, one-way ANOVA was used followed by Tukey’s or Dunnett’s T3 post hoc tests in the case of homogeneous or heterogeneous variance data, respectively. When data did not fit a normal distribution, the non-parametric Kruskal–Wallis test, followed by Mann–Whitney test, was used. Statistical analyses were performed using SPSS Statistics version 20 (IBM, Armonk, NY, USA). Results were presented as mean ± SEM in tables and mean + SEM in figures. *p* < 0.05 was considered to indicate a statistically significant difference. Graphs were generated using GraphPad Prism version 6.00 for Windows (GraphPad Software, La Jolla, CA, USA, www.graphpad.com).

## Figures and Tables

**Table:** 

A	EPA	DHA	LA	ALA
*cebpa*	0.54 ± 0.144 *	0.63 ± 0.079	0.71 ± 0.077	0.73 ± 0.055
*pparg*	0.51 ± 0.109 *	0.66 ± 0.044	0.70 ± 0.128	0.81 ± 0.094
*lxr*	0.91 ± 0.112	0.96 ± 0.064	0.88 ± 0.068	0.95 ± 0.046
*fas*	0.69 ± 0.138	0.80 ± 0.109	0.74 ± 0.100	0.89 ± 0.107
*lpl*	0.97 ± 0.119	1.02 ± 0.066	0.98 ± 0.067	1.03 ± 0.059
*hsl*	1.12 ± 0.040	1.07 ± 0.059	0.94 ± 0.065	1.06 ± 0.051
*cd36*	0.70 ± 0.197	0.90 ± 0.091	0.83 ± 0.034	0.93 ± 0.067
*fatp1*	1.38 ± 0.158 ^a.b^	1.29 ± 0.029 ^b^	1.64 ± 0.116 *^a^	1.78 ± 0.077 *^a^
*fabp11a*	1.27 ± 0.160	1.13 ± 0.155	1.14 ± 0.138	1.11 ± 0.106
*abca1 *	0.85 ± 0.353	0.84 ± 0.057	0.80 ± 0.079	0.92 ± 0.093

**Table:** 

B	EPA + DHA	EPA + LA	EPA + ALA	DHA + LA	DHA + ALA	LA + ALA
*cebpa*	1.28 ± 0.208	0.78 ± 0.141	0.72 ± 0.091	0.80 ± 0.173	0.91 ± 0.079	0.84 ± 0.070
*pparg*	0.24 ± 0.027 *	0.31 ± 0.035 *	0.30 ± 0.036 *	0.41 ± 0.057 *	0.42 ± 0.046 *	0.42 ± 0.092 *
*lxr*	0.71 ± 0.040 *	1.06 ± 0.151	0.93 ± 0.126	0.94 ± 0.209	0.93 ± 0.078	0.87 ± 0.044
*fas*	1.23 ± 0.117	1.45 ± 0.200	1.29 ± 0.252	1.05 ± 0.113	1.28 ± 0.214	1.34 ± 0.222
*lpl*	0.95 ± 0.128	1.06 ±0.134	0.95 ± 0.106	1.09 ± 0.198	0.94 ± 0.118	0.79 ± 0.112
*hsl*	1.2 ± 0.159	1.21 ± 0.145	1.09 ± 0.116	1.13 ± 0.234	1.13 ± 0.051	1.02 ± 0.099
*cd36*	1.08 ± 0.051	1.46 ± 0.236	1.38 ± 0.197	1.02 ± 0.157	1.25 ± 0.122	1.33 ± 0.219
*fatp1*	1.04 ± 0.064 ^a^	1.73 ± 0.178 *^b^	1.62 ± 0.160 ^ab^	1.47 ± 0.169 ^ab^	1.41 ± 0.105 ^ab^	1.76 ± 0.180 *^b^
*fabp11*	1.02 ± 0.078	1.32 ± 0.152	1.09 ± 0.054	1.12 ± 0.176	1.05 ± 0.123	1.25 ± 0.129
*abca1*	1.32 ± 0.189	1.52 ± 0.296	1.25 ± 0.184	1.2 ± 0.337	1.1 ± 0.170	1.09 ± 0.203

**Table 2:** Rainbow trout primers used for real-time quantitative PCR. F, forward primer; R, reverse primer; Ta, annealing temperature; Acc. Num., GenBank accession number.

Gene	Primer Sequence (5’→3’)	Ta (°C)	Acc. Num.
*Cebpa*	F: TGTGGCGATAAAGCAAGAGC	57	DQ423469.1
	R: CTGGTGGGAATGGTGGTAGG		
*Cebpb*	F: CACAAAGTGCTGGAACTGGC	60	FR904306.1
	R: TGGCACAGCGATAAATGGGT		
*Ppara*	F: CTGGAGCTGGATGACAGTGA	54	AY494835
	R: GGCAAGTTTTTGCAGCAGAT		
*Pparb*	F: CTGGAGCTGGATGACAGTGA	59	AY356399.1
	R: GTCAGCCATCTTGTTGAGCA		
*Pparg*	F: GCCAGTACTGTCGCTTTCAG	60	HM536192.1
	R: TCCATAAACTCAGCCAGCAG		
*Lxr*	F: TGCAGCAGCCGTATGTGGA	62	NM_001159338
	R: GCGGCGGGAGCTTCTTGTC		
*Rxr*	F: AAAGAGCGCAGTGAGAACGA	55	AJ969439.1
	R: TGTAGGTCTCGGTCTTGGGT		
*Fas*	F: GAGACCTAGTGGAGGCTGTC	54	tcaa0001c.m.06_5.1.om.4
	R: TCTTGTTGATGGTGAGCTGT		
*Lpl*	F: TAATTGGCTGCAGAAAACAC	59	AJ224693
	R: CGTCAGCAAACTCAAAGGT		
*hsl*	F: AGGGTCATGGTCATCGTCTC	58	TC172767
	R: CTTGACGGAGGGACAGCTAC		
*cd36*	F: CAAGTCAGCGACAAACCAGA	62	AY606034
	R: ACTTCTGAGCCTCCACAGGA		
*fatp1*	F: AGGAGAGAACGTCTCCACCA	60	CA373015
	R: CGCATCACAGTCAAATGTCC		
*fabp11*	F: CATTTGAGGAGACCACCGCT	60	NM_001124713.1
	R: ACTTGAGTTTGGTGGTACGCT		
*abca1*	F: CAGGAAAGACGAGCACCTTC	58	TC169876
	R: TCTGCCACCTCACACACTTC		
*18s*	F: GGCGCCCCCTCGATGCTCTTA	65	AF308735.1
	R: CCCCCGGCCGTCCCTCTTAAT		
*ef1a*	F: TCCTCTTGGTCGTTTCGCTG	58	AF498320
	R: ACCCGAGGGACATCCTGTG		
*b-actin*	F: ATCCTGACAGAGCGCGGTTACAGT	61	AJ438158
	R: TGCCCATCTCCTGCTCAAAGTCAA		
*Ub*	F: ACAACATCCAGAAAGAGTCCAC	58	AB036060
	R: AGGCGAGCGTAGCACTTG		

## Abbreviations

LC-PUFA	long-chain polyunsaturated fatty acids
EPA	eicosapentaenoic acid
DHA	docosahexaenoic acid
FA	Fatty acid
LA	Linoleic acid
ALA	Alpha-linolenic acid
PPAR	peroxisome proliferator-activated receptor
FAS	fatty acid synthase
CD36	Fatty acid translocase/cluster of differentiation 36
FO	fish oil
VO	vegetable oil
OA	oleic acid
PA	palmitic acid
AA	arachidonic acid
C/EBP	CCAAT/enhancer binding protein
HSL	hormone sensitive lipase
FABP11	fatty acid binding protein 11
FATP1	fatty acid transporter protein 1
LD	lipid droplets
ORO	Oil red O
LXR	liver X receptor
NEFA	non-esterified fatty acids
RXR	retinoid X receptor
LPL	lipoprotein lipase
Ef1a	elongation factor 1 alfa
Ub	ubiquitin
β-actin	beta-actin
ABCA1	ATP-binding cassette transporter 1
